# Inducing hypertension in *Myh11*^R247C/R247C^ mice triggers aortic dissections with increased focal adhesion kinase signaling

**DOI:** 10.3389/fcvm.2025.1492768

**Published:** 2025-02-14

**Authors:** Callie S. Kwartler, Shanzhi Wang, Zhen Zhou, Pujun Guan, Yang Yu, Xue-Yan Duan, Theodore Zhang, Jiyuan Chen, Elaine C. Davis, Dianna M. Milewicz

**Affiliations:** ^1^ Division of Medical Genetics, Department of Internal Medicine, The University of Texas Health Science Center at Houston, Houston, TX, United States; ^2^ Department of Anatomy and Cell Biology, McGill University, Montreal, QC, Canada

**Keywords:** myosin heavy chain, focal adhesion, elastin-contractile unit, thoracic aortic dissection, vascular disease, aortic dissection, smooth muscle cell (SMC)

## Abstract

**Objective:**

We sought to determine if hypertension in combination with a “variant of uncertain significance” that disrupts protein function, *MYH11* p.Arg247Cys, would induce aortic dissections in a mouse model.

**Approach and results:**

Administration of L-NAME via drinking water and a high salt diet increased blood pressure in WT and *Myh11^R247C/R247C^* mice and triggered type A dissections with cardiac tamponade in 20% of the *Myh11^R247C/R247C^* mice. *Myh11^R247C/R247C^* aortas have aberrant smooth muscle contractile unit-elastin connections by transmission electron microscopy, along with increased focal adhesion signaling at baseline, which further increases with hypertension.

**Conclusion:**

Gene-environment interactions trigger aortic dissections in *Myh11^R247C/R247C^* mice.

## Introduction

Acute ascending aortic dissections (ADs; Stanford classification type A) cause sudden death in up to half of afflicted individuals, and these sudden deaths are primarily due to retrograde dissection of the blood from the aortic intimal tear at the sinotubular junction into the pericardial sac, causing pericardial tamponade (1). Typically, enlargement of the ascending aorta or root precedes the AD, and prophylactic surgery of these aneurysms is recommended when the diameter reaches >5.0 cm to prevent AD (2). However, studies on patients presenting with acute ADs determined that approximately 50% of dissections occur with no enlargement or at diameters <5.0 cm in diameter (3).

The major risk factors for ADs are genetic variants and increased biomechanical force on the aorta, primarily due to hypertension (HTN) (4). Pathogenic variants in *MYH11*, which encodes the smooth muscle cell (SMC)-specific isoform of myosin heavy chain, predispose to ADs, and these rare variants are primarily in-frame deletions in the coiled-coil domain, which disrupt thick filament formation (5, 6). *MYH11* and other mutant genes that confer a highly penetrant risk for AD disrupt proteins in a structural element in the aorta, termed the elastin-contractile unit (ECU), which links extensions from the elastin fibers to SMC contractile units through cell surface focal adhesions (FAs) (7). We have also identified a *MYH11* rare variant of unknown significance (VUS), *MYH11* p.Arg247Cys, in patients with thoracic aortic disease, and disruption of the corresponding amino acid in *MYH7* causes familial hypertrophic cardiomyopathy (8, 9). We previously showed that *Myh11*^R247C/R247C^ mice do not form aneurysms despite decreased aortic contraction, and explanted *Myh11*^R247C/R247C^ SMCs have increased focal adhesion kinase (FAK) signaling (9). Increased FA signaling has also been shown in the *Acta2*^−/−^ mouse model, which develops aortic aneurysms, and single cell transcriptomics of *Fbn1*^mgr/mgr^ mice and angiotensin II-infused mice implicate FA signaling in ADs (10–12). When a second genetic hit, loss of one allele of *Acta2*, was introduced in *Myh11*^R247C/R247C^ mice, the mice developed thoracic aortic enlargement, supporting that *MYH11* p.Arg247Cys is a risk allele for thoracic aortic disease (13). Here, we report that HTN leads to death due to acute ADs in the absence of aortic enlargement in 20% of *Myh11*^R247C/R247C^ mice, indicating that this is also a risk allele for acute ADs.

## Methods

### Hypertensive model in mice

Wild-type (WT) and *Myh11*^R247C/R247C^ mice on 129S8/SvEv and C57BL/6 mixed background were produced as previously reported (9). Littermate controls were used for all experiments to ensure equal percentages of the two strains between treatment and control groups. All protocols were approved by the Animal Welfare Committee at the University of Texas Health Science Center at Houston.

Since incidence of thoracic aortic disease is substantially higher in men (4), only male mice were treated and assessed in this study. *Myh11*^R247C/R247C^ and WT littermates were randomized into HTN or untreated groups. High salt diet (8% NaCl diet from Harlan Laboratories) and L-Nω-nitroarginine methyl ester (L-NAME, Cayman Chemical) were used to induce HTN in male mice [treatment initiated at 8–9 weeks of age except for the transmission electron microscopy (TEM) studies]. L-NAME was dissolved at 3.0 g/L in the drinking water, and fresh water was prepared and replaced daily.

Blood pressure was alternatively increased in mice through intraperitoneal injection of 0.05 mg norepinephrine (NE) on the evening of day 1, then injection with 0.025 mg norepinephrine in the morning and evening on day 2.

Additional methods are available in the supplement.

## Results

### Hypertension causes aortic root dissection in *Myh11^R247C/R247C^* mice

**Figure 1:**
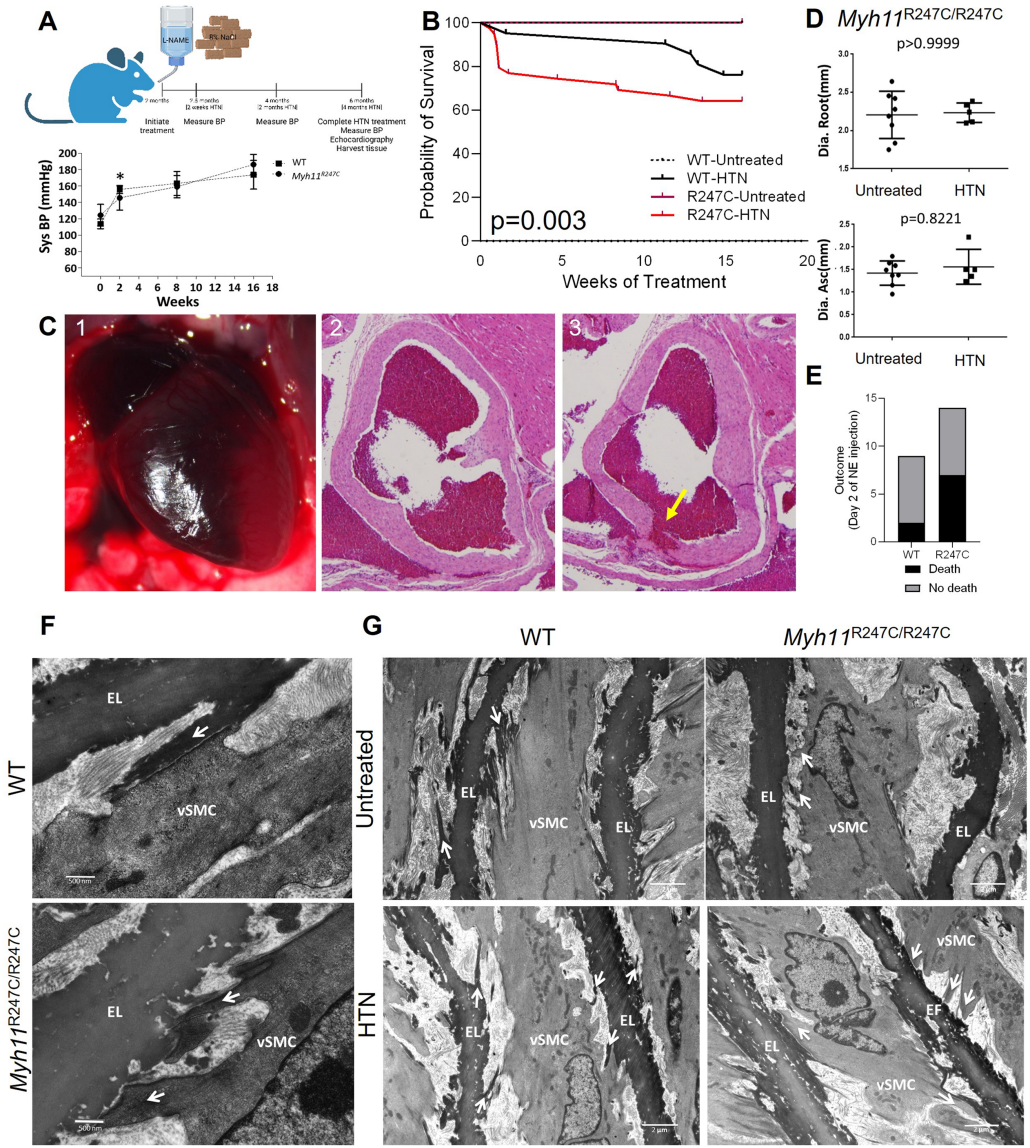
HTN in *Myh11*^R247C/R247C^ mice induces aortic dissections. **(A)** Top panel shows a schematic of the experiment design: L-NAME and high salt diet were initiated in 2 month old mice. Bottom panel shows tail cuff blood pressure measurements indicating significantly increased systolic and diastolic blood pressures within two weeks after initiation of L-NAME and high salt diet treatment in both WT and *Myh11*^R247C/R247C^ mice. **(B)** Kaplan–Meier survival analysis shows reduced survival over sixteen weeks of HTN induction in hypertensive *Myh11*^R247C/R247C^ mice compared with hypertensive WT mice or normotensive mice (*n* = 16 untreated WT, *n* = 27 untreated *Myh11*^R247C/R247C^, *n* = 21 HTN WT, *n* = 39 HTN *Myh11*^R247C/R247C^). **(C)** Example necropsy on *Myh11*^R247C/R247C^ mouse that did not survive shows pericardial tamponade (1). Sectioning through the aortic root shows a tear in the intimal layer, i.e., dissection (3); an adjacent section does not show the dissection (2). **(D)** Echocardiography after four months of HTN treatment reveals no increase in aortic diameter in either WT or *Myh11*^R247C/R247C^ mice after HTN induction. Dots represent individual mice, lines represent the mean ± standard deviation. Statistical comparison was measured by non-parametric Mann–Whitney analysis. **(E)** Injection of norepinephrine (NE) induced sudden death within two days in 6/14 *Myh11*^R247C/R247C^ mice compared with 2/9 WT mice. **(F)** Transmission electron microscopy (TEM) images of the four-week-old aorta (untreated) show aberrant structure of the elastin-contractile unit in *Myh11*^R247C/R247C^ aortas with increased distortion of *Myh11*^R247C/R247C^ SMCs after HTN. Arrows indicate projections linking smooth muscle cells (vSMC) with elastin fibers (EL); in WT tissue those projections initiate from the EL, while in *Myh11*^R247C/R247C^ aortas those projections initiate from vSMC (*n* = 2 aortas per genotype, minimum of 3 visual fields per aorta). **(G)** TEM images of untreated four-week-old aortas and specimen at six weeks of age after two weeks of HTN confirm increased distortion (*n* = 2 aortas per genotype and treatment condition, minimum of 3 visual fields per aorta).

We previously demonstrated decreased contraction of aortic segments but no aortic enlargement or spontaneous death in the *Myh11^R247C/R247C^* mice (9). To increase biomechanical forces on the aorta, 8-week-old male WT and *Myh11^R247C/R247C^* mice were subjected to a high salt diet and the nitric oxide synthase inhibitor L-NAME. Littermates were randomized into HTN or untreated groups. Since incidence of thoracic aortic disease is substantially higher in males, only male mice were treated and assessed in this study (4). The treatment significantly increased blood pressure within two weeks to the same extent in *Myh11^R247C/R247C^*and WT mice (Figure 1A). Over the sixteen weeks after HTN induction, sudden deaths occurred in 36% of the *Myh11^R247C/R247C^* mice (14 deaths/39 mice) while only five WT mice died (5 deaths/21 mice), and no untreated mice of either genotype died (*p* = 0.003 by Kaplan–Meier analysis) (Figure 1B). Nine *Myh11^R247C/R247C^* deaths compared with just one WT death occurred in the first two weeks after HTN induction. Necropsy of *Myh11^R247C/R247C^* mice identified blood in the pericardial sac by visual inspection and histology (Figure 1C; Supplementary Figure 1A). Serial sectioning from the aortic valve to the ascending aorta identified intimal tears at the sinotubular junction as the cause of the pericardial tamponade, thus mimicking ADs in patients. (Figure 1C; Supplementary Figure 1). No pericardial tamponade or intimal tears were observed in the dead WT mouse. Echocardiography identified no enlargement of the aortic root or ascending aorta in the surviving *Myh11^R247C/R247C^* mice out to six months of age, four months after HTN induction (Figure 1D; Supplementary Figure 2A). After echocardiography, aortas were fixed and paraffin embedded; hematoxylin and eosin (HE) staining confirmed increased thickness of the medial layer in the hypertensive compared with untreated *Myh11^R247C/R247C^* mice (Supplementary Figure 2B). To confirm that HTN was responsible for the deaths in the *Myh11^R247C/R247C^* mice, HTN was induced using intraperitoneal injections of NE, leading to a systolic blood pressure increase of 50 mmHg within 10 min of the injection. Two of nine WT mice compared with seven of fourteen mutant mice died suddenly on day two of NE injections, and pericardial tamponade was observed in the *Myh11^R247C/R247C^* mice without enlargement of the aorta (*p* value = 0.2, Figure 1E; Supplementary Figure 3). Thus, other methods to induce HTN also lead to aortic dissections in *Myh11^R247C/R247C^* mice.

### Aberrant structure and signaling of the SMC elastin-contractile unit in the hypertensive *Myh11^R247C/R247C^* mice

TEM of 4-week-old WT ascending aortas confirmed previous findings of the structure of the aortic media: specifically, oblique extensions from the elastic fibers linked to FAs on the surface of SMCs, indicating formation of intact ECUs (Figure 1F) (14). In contrast, ECUs were structurally abnormal in 4-week-old *Myh11^R247C/R247C^* mice; the oblique extensions from the elastin fiber were not present but rather cellular extensions from the SMCs attached to the elastin fiber (Figure 1F). Moreover, HTN treatment for two weeks induced distortion of the elastin extensions but little distortion of the SMCs in the WT aortas, whereas the SMCs are distorted with HTN in the *Myh11^R247C/R247C^* aortas (Figure 1G). It is important to note that the untreated mice were imaged at four weeks of age, while the HTN mice were imaged at six weeks of age, and therefore we cannot rule out that age-related changes are responsible for the differences observed in the HTN groups.

**Figure 2:**
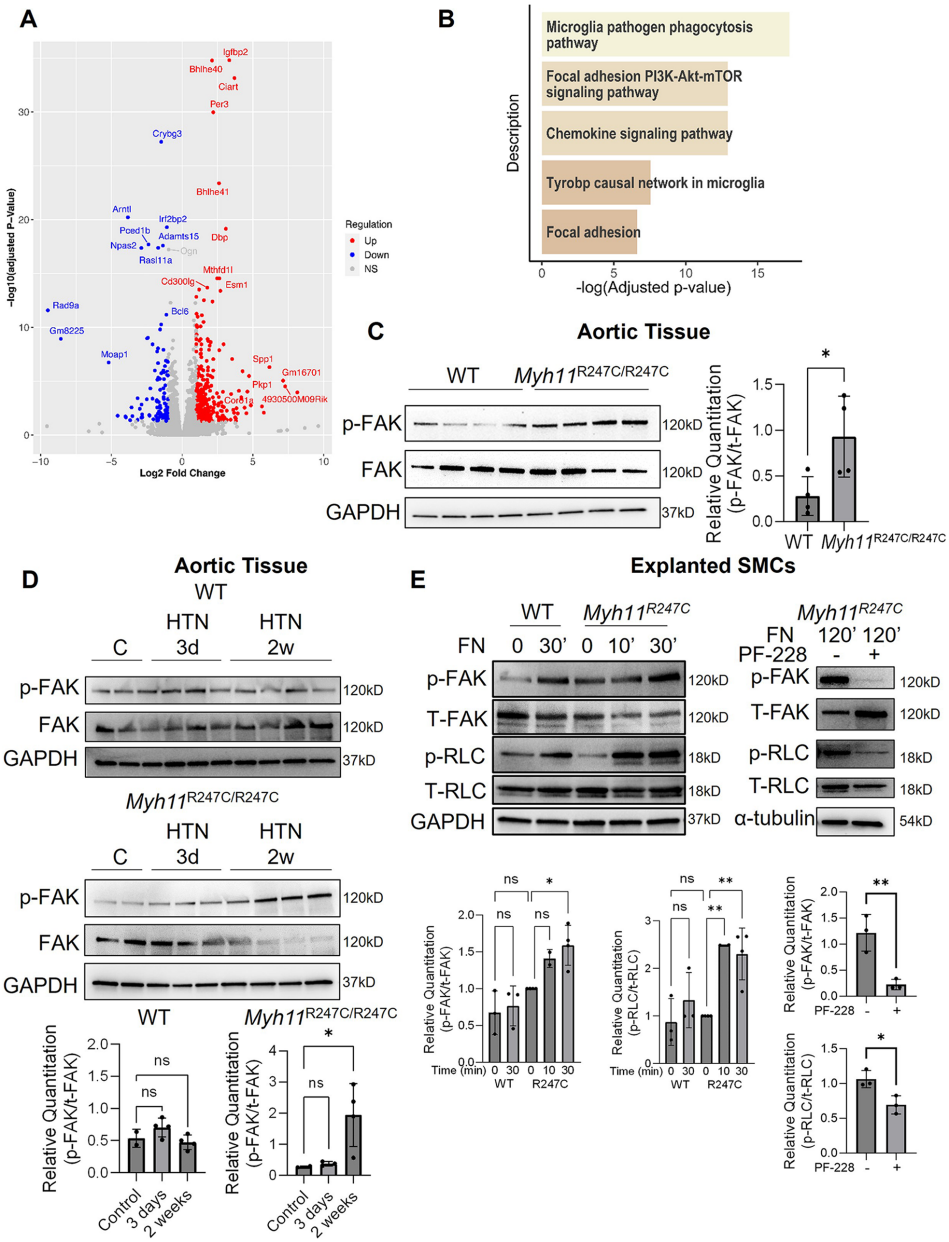
Aberrant ECU structure and FA signaling in *Myh11*^R247C/R247C^ aortas. **(A,B)** Bulk RNA-sequencing analysis on descending aortic tissue revealed differentially expressed genes **(A)**, and GSEA analysis of those genes revealed the top five upregulated pathways in the *Myh11*^R247C/R247C^ mice after HTN include focal adhesion-related signaling **(B)**. **(C)** Western blot analysis confirms increased FAK activation in *Myh11*^R247C/R247C^ aortic tissue at baseline compared to WT tissue (without HTN). **(D)** Further analysis of WT or *Myh11*^R247C/R247C^ tissue shows increased FAK activation within two weeks of HTN induction in *Myh11*^R247C/R247C^ but not WT aortas. **(E)** Explanted SMCs from *Myh11*^R247C/R247C^ have increased activation of FAK and increased phosphorylation of myosin regulatory light chain (RLC) after activation of the focal adhesions by short-term addition of fibronectin (FN) compared with SMCs explanted from littermate controls. The right panel shows *Myh11*^R247C/R247C^ SMCs treated with FN for two hours with or without the FAK inhibitor PF-228. All blot quantitations are shown; each dot represents an individual mouse **(C,D)** or an independently-run experiment **(E)** Statistical analysis was performed via two-tailed student's *t*-test **(C)** or via one-way ANOVA with Dunn's post-test **(D,E)**. *p < 0.05, **p < 0.005, HTN, hypertension; vSMC, smooth muscle cell; EF, elastin fiber; FAK, focal adhesion kinase; RLC, myosin regulatory light chain.

The distortion of the abnormally formed ECUs in aortic SMCs with HTN in the *Myh11^R247C/R247C^* mice suggests that mechanosensing pathways downstream of FAs may be activated. Global transcriptional changes in the *Myh11^R247C/R247C^* descending aortas with and without HTN induction for two weeks were assessed using RNA-sequencing; 644 upregulated and 817 downregulated genes were identified (Figure 2A). GSEA analysis using WikiPathways identified five upregulated and no downregulated pathways; of the five upregulated pathways, two involve FA signaling (Figure 2B; Supplementary Figure 2). We previously showed that *Myh11^R247C/R247C^* SMCs explanted from the ascending aorta had altered FAs and increased phosphorylation at tyrosine 397 of FAK (p-FAK) levels when compared to WT SMCs (9). Here, we found that *in vivo* immunoblot analyses confirm increased levels of p-FAK in the *Myh11^R247C/R247C^* aortas at baseline (Figure 2C), and levels of p-FAK increase further after two weeks of HTN induction in *Myh11^R247C/R247C^* aortas but not in WT aortas (Figure 2D). Since phosphorylation of the RLC cannot be accurately assessed *in vivo* due to its transient nature (15), these levels were assessed in explanted SMCs. Exposure to soluble fibronectin (FN) activates FAs in the SMCs, and the *Myh11^R247C/R247C^* SMCs have higher levels of p-FAK and p-RLC than WT SMCs after 30 min of FN exposure (Figure 2E). Importantly, a FAK inhibitor, PF-228, significantly attenuated phosphorylation of both FAK and RLC 2 h after FN treatment in *Myh11^R247C/R247C^* SMCs (Figure 2E).

## Discussion

Although genetic evidence supports that disruption of SMC force generation is a primary driver of ADs (16, 17), *Myh11^R247C/R247C^* mice have decreased aortic contraction but do not form aneurysms (9). However, the *Myh11^R247C^* allele does cause aortic enlargement in combination with a second genetic “hit”, loss of one allele of *Acta2 (*13). Here, increasing systolic blood pressure using L-NAME and high salt diet rapidly induced sudden death due to ADs and pericardial tamponade in 23% of *Myh11^R247C/R247C^* mice without evidence of aortic enlargement. HTN is the major risk factor for patients with thoracic ADs (4), and the results presented here indicate that sudden increases in blood pressure can induce ADs without aortic enlargement. Thus, this study is the first to demonstrate that sudden onset of HTN in conjunction with a VUS in *MYH11* confers a low penetrant risk for acute ADs.

We further show that ECUs have an aberrant structure in *Myh11^R247C/R247C^* aortas, suggesting SMC contraction is required for the formation of extensions from elastin to SMCs. Additionally, transcriptomic data and immunostaining of aortic lysates indicate increased FAK signaling at baseline in the *Myh11^R247C/R247C^* aortas, which increases further with the induction of HTN in the *Myh11^R247C/R247C^* aortas. Importantly, these conclusions are based on analyses of mice that did not dissect, so although our data indicate that HTN increases FA-related signaling, other pathways driving dissection may have not been identified due to survival bias. Thus, these data suggest that the loss of SMC contractile function in *Myh11^R247C/R247C^* aortas increases signaling through FAs, and most likely through increases in downstream p-RLC levels and augmented actinomyosin motor function, which we hypothesize may compensate for the defective myosin function, thus preventing aortic disease. With the onset of HTN, the acute and excessive activation of FAK is predicted to further drive downstream actinomyosin motors but also activate additional pathways. Because few *Myh11^R247C/R247C^* mice die after the first two weeks of HTN treatment, we hypothesize that chronic HTN leads to adaptive changes that prevent further deaths. An important limitation of the current study is that only male mice were analyzed; further work is needed to establish whether these effects are sex-dependent. Thus, these data illustrate the interaction between a risk variant and HTN in triggering acute ADs and suggest a hypothesis that compensatory changes in the aorta occur in response to the *Myh11* mutation that prevent both aneurysm formation at baseline and further AD deaths with chronic HTN.

## Data Availability

The original contributions presented in the study are included in the article/supplementary material, further inquiries can be directed to the corresponding author.
