# Supplementary material for: Inducing hypertension in Myh11R247C/R247C mice triggers aortic dissections with increased focal adhesion kinase signaling
Source: Front Cardiovasc Med. 2025 Feb 14;12:1492768. doi: 10.3389/fcvm.2025.1492768 (PMC11868086; doi:10.3389/fcvm.2025.1492768)
Supplement: Supplementary file 1 [file Datasheet1.docx]

**Inducing Hypertension in *Myh11^R247C/R247C^* Mice Increases Focal Adhesion Signaling and Triggers Aortic Dissections**

Callie S. Kwartler^1*^, Shanzhi Wang^1*^, Zhen Zhou^1*^, Pujun Guan^1^, Yang Yu^1^, Xue-Yan Duan^1^, Jiyuan Chen^1^, Elaine C. Davis^2^, Dianna M. Milewicz^1^

Supplemental Information

**Supplemental Methods**

*Blood pressure monitoring.*

Systolic and diastolic blood pressures of conscious mice were measured using a tail cuff blood pressure analyzer designed with volume-pressure recording (VPR) technology (Model Coda, Kent Scientific Technology). Body temperatures of the mice were maintained at 37°C by a heated platform. The blood pressure was monitored for 60 cycles. Final values were obtained after 15 consecutive readings and the values with blood volume larger than 20ul were used for calculation. The measurements were taken for three consecutive days and the mean value of the readings for every day per mouse were averaged and taken as the single blood pressure measurement for that animal. This protocol was repeated in 8-week-old mice prior to initiation of hypertension (**HTN**), and again after 2 weeks, 8 weeks, or 16 weeks of the L-N𝝎-nitroarginine methyl ester (**L-NAME**) plus high salt diet regimen.

*Characterization of aortic dissection*

All mice enrolled in the trial were checked daily first thing in the morning, and any mouse found dead was subjected to immediate necropsy. First, assessment was made whether there was frank blood in any cavity or any obvious cause of death. In all cases, the heart with ascending aorta attached was dissected out and fixed in 10% formalin for 24 hours. Following fixation, the heart was cut into two pieces along the bottom line of atria. Both parts were subjected to routine paraffin embedding and serially sectioned through the entire tissue at 4 micrometers per section. The slides were stained with hematoxylin and eosin (**HE**) method and pictures were taken with Nikon microscope. To confirm an aortic dissection event, any abnormality observed in one section was examined in the adjacent sections on either side as well. To avoid technical issues of necropsy sectioning, the same abnormality had to be observed in at least three consecutive sections to conclude the phenotype. Medial thickness was quantified on HE stained aortic sections using ImageJ software, with 3 measurements per section of at least 3 sections quantified for each aorta.

*Echocardiography and Doppler studies*

A separate cohort of mice were put on the L-NAME and high salt diet treatment to induce HTN and were followed long-term for echocardiography studies. Ultrasound was performed with a Vevo 770 imaging system at 8 weeks using a 40 MHz 704 probe (VisualSonics, Toronto, Canada). Mice were anesthetized by inhalation of isoflurane (2.5%) while ECG and respirations were continuously monitored on a warmed platform. The anterior chest was denuded using depilatory cream prior to application of ultrasound gel and imaging. Images were then exported to Sante DICOM Editor software (SanteSoft LTD, Athens Greece) for further analysis. The dimensions of the ascending aorta (in mm) were measured in a single plane using right parasternal views at two levels (aortic root and ascending aorta). All scanning and analysis was performed by an experienced ultrasound technician who was blinded to the mouse genotype and treatment. Data for the latest timepoint assessed (at 6 months of age, 4 months after HTN induction) are included in the manuscript.

*Transmission electron microscopy*

Transmission electron microscopy (**TEM**) was performed on 4-week-old WT and *Myh11*^R247C/R247C^ aortas and on 6-week-old WT and *Myh11*^R247C/R247C^ after two weeks of L-NAME and high salt diet aortas (n=3 per genotype per timepoint). Mice were perfused with PBS, followed by 3% glutaraldehyde in 0.1% sodium cacodylate (pH=7.4) to fix the tissues. Ascending aortas were dissected out and fixed further overnight, then treated sequentially with osmium tetroxide, tannic acid and uranyl acetate and dehydrated and embedded in Epon as previously described (1). Sixty-nanometer sections were counterstained with 7% methanolic uranyl acetate and lead citrate and observed using Tecnai 12 transmission electron microscope operating at 120 kV.

*Bulk RNA sequencing*

Eight-week-old male *Myh11^R247C/R247C^* mice were subjected to HTN or control treatment for two weeks (n=3 per group, RNA was separately prepped and sequenced from each mouse). Total RNA were extracted from descending thoracic aortas with RNeasy kit (Qiagen) followed by library preparation and sequencing at paired ends at 150-bp read length on NovaSeq 6000, Illumina. Raw reads were trimmed using Fastp v0.19.5, and trimmed reads were then aligned to the mouse reference genome (mm10) using Hisat2 v2.1 with default parameters. SAMtools v1.9 was used to extract unique mapped reads and remove duplicated reads. Quantification of genes annotated in GRCm38.99 from Ensembl database was estimated using String-Tie v1.3.4. Genes with normalized expression value, fragments per kilobase of exons per million reads mapped (FPKM), >1 in at least one sample were considered. Read counts that were measured for each gene using featureCounts v1.5.1 were used as the input to DESeq2 for differential expression analysis with adjusted *P*-value <0.05 and fold change >2. Gene Set Enrichment Analysis (GSEA) was conducted by clusterProfiler (ver: 4.8) using differential expression data, where genes were ranked by log2 fold changes. The WikiPathways annotation (ver:20240210) was used, and the gene set size limits were between 30 and 400 to exclude overly broad or confined gene sets.

*Western blot analysis of aortic tissue*

Descending aortic tissues from WT and *Myh11*^R247C/R247C^ mice at 8 weeks of age for baseline blots (Figure 2B) or from mice at the designated timepoints after initiation of L-NAME and high salt diet (Figure 2C) were isolated and flash-frozen in liquid nitrogen. None of the tissues utilized for Western blot had signs of aortic dissection at the time of harvest. Tissues were manually ground using a mortor and pestle, then lysed in RIPA lysis buffer supplemented with protease inhibitor cocktail (P8340, Sigma) and phosphatase inhibitor cocktails 2 and 3 (Sigma). After protein quantification (Bio-Rad), 10-15 ug protein per sample was loaded and run on 4-20% SDS-PAGE gel (Bio-Rad) and transferred to PVDF membrane. Membranes were blocked with 5% milk in TBST (20mM Tris, 150mM NaCl, 0.05% Tween 20, pH 7.6), then incubated with primary antibody in 3% milk in TBST overnight at 4°C. Antibodies against phosphorylated FAK (**p-FAK**, #3283), total FAK (**t-FAK**, #3285), and GAPDH (#2118) are from Cell Signaling Technologies; dilution of GAPDH antibody is 1:10,000, for all other primary antibodies dilution is 1:1000. Anti-Rabbit IgG secondary antibody (BioRad) was diluted 1:2000 in 1 x TBST with 3% milk TBST solution and incubated at room temperature for one hour.

*Aortic smooth muscle cell cultures and immunoblotting*

Independent cell lines were explanted from the ascending aortas of wild type and *Myh11*^R247C/R247C^ mice, using aortas pooled from three to five mice per genotype per explant as previously described (2). Smooth muscle cells (**SMCs**) were cultured in complete media (SmBM Medium (Lonza Cambrex) including 20% fetal bovine serum (Gibco), 1X Antibiotic/Antimycotic, 20mM L-Glutamine, 10mM sodium pyruvate, 20mM HEPES (all Gibco), and growth factors from Cambrex SMBM bullet kit (fibroblast growth factor, insulin and endothelial growth factor).

Aortic SMCs (2 x105 cells) were plated in 60 mm dishes for 16 hours, and then the cells serum starved (1% SMC media) for 24 hours. Fibronectin (Sigma) 60ug was diluted in 700uL 1X DPBS and 5ug/cm^2^ of fibronectin solution was added to the culture media for the indicated time periods. After incubation, the cells were washed with cold PBS two times, then 60 uL lysis buffer (RIPA plus protease and phosphatase inhibitors as described above) was added into dishes. The whole dishes were snap-frozen in liquid nitrogen and stored at -80 degree. After thaw, cells were scraped in the lysis buffer and cell lysates were collected, quantified (Bio-Rad), and run on SDS-PAGE gels as described above. All experiments were using passage-matched WT and *Myh11*^R247C/R247C^ SMCs. All studies were performed on SMCs earlier than passage 5. Antibodies against phosphorylated FAK (**p-FAK**, #3283), total FAK (**t-FAK**, #3285), phosphorylated regulatory light chain (**p-RLC**, #3674), total regulatory light chain (**t-RLC**, #3672), and GAPDH (#2118) are from Cell Signaling Technologies; dilution of GAPDH antibody is 1:10,000, for all other primary antibodies dilution is 1:1000.

*Statistical analyses*

All data were analyzed in Graphpad Prism software (version 7.0). Survival analysis was performed using Kaplan-Meier statistics. For systolic blood pressure analysis, blood pressure increases were assessed by comparing baseline blood pressure with later timepoints within each genotype using a two-tailed student’s *t-*test. For echocardiography and Western blot quantitation analyses, data were tested for normality and analyzed via student’s *t-*test or nonparametric Mann-Whitney analysis when comparing two groups, and by one-way ANOVA or nonparametric Kruskal-Wallis analysis when comparing more than two groups. For all *in vivo* analyses, each mouse was counted as a separate data point. For cell culture experiments, independent experimental replicates were counted as separate data points. In all cases, p<0.05 was counted as statistically significant. Details about the specific test and n numbers for each experiment are included in the figure legends.

**References**

1. Davis EC. Smooth muscle cell to elastic lamina connections in developing mouse aorta. role in aortic medial organization. Lab Invest. 1993 January 01;68(1):89-99.

2. Kwartler C, Zhou P, Kuang S, Duan X, Gong L, Milewicz D. Vascular smooth muscle cell isolation and culture from mouse aorta. Bio-protocol. 2016;6(23).

**
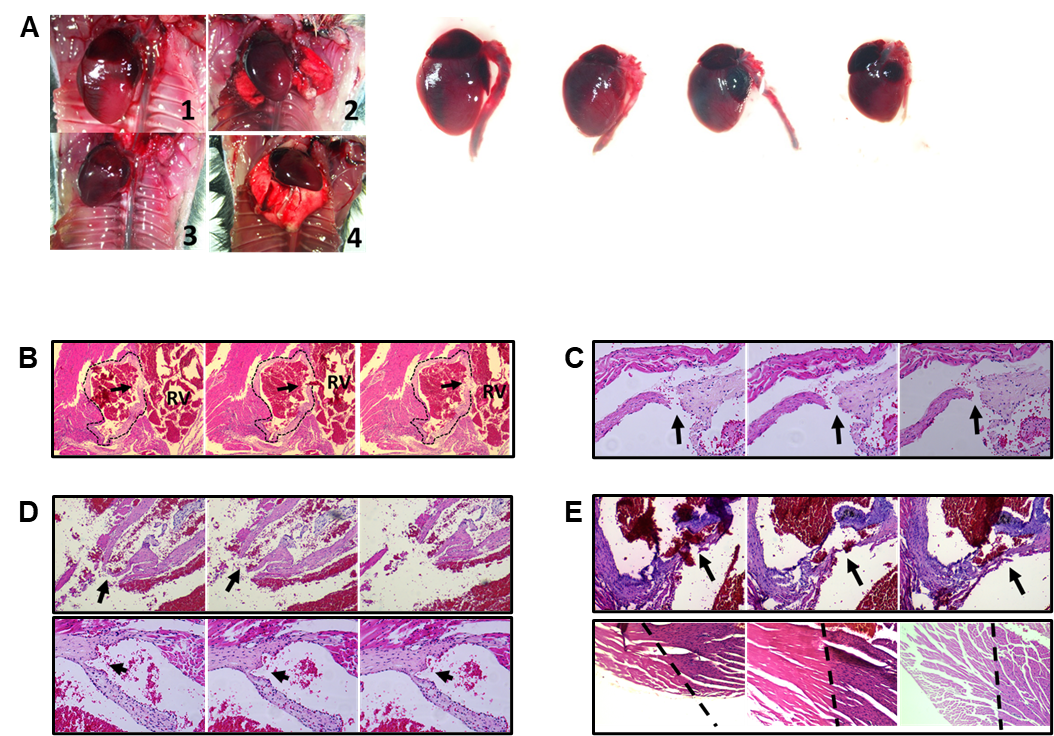
Supplemental Figures and Legends**

**Supplemental Figure 1.** A) Gross images showing lack of aortic enlargement and pericardial tamponade in *Myh11*^R247C/R247C^ mice mice that died after L-NAME/High salt. B) Histology sections from heart 1 shows an aortic root dissection with blood flow from the aorta into the right ventricle (RV). C) Histology sections from heart 2 show aortic rupture next to the aortic valve. D) Histology sections from heart 3 show aortic rupture (top panels) with a partially torn aortic valve (bottom panels). E) Histology sections from heart 4 show aortic root dissection with blood flow into the ventricle (top panels). In this mouse, there was also a loss of the cardiomyocyte structure at the left/right ventricle interface (dotted line) suggesting involvement of the coronary artery (bottom panels).

**
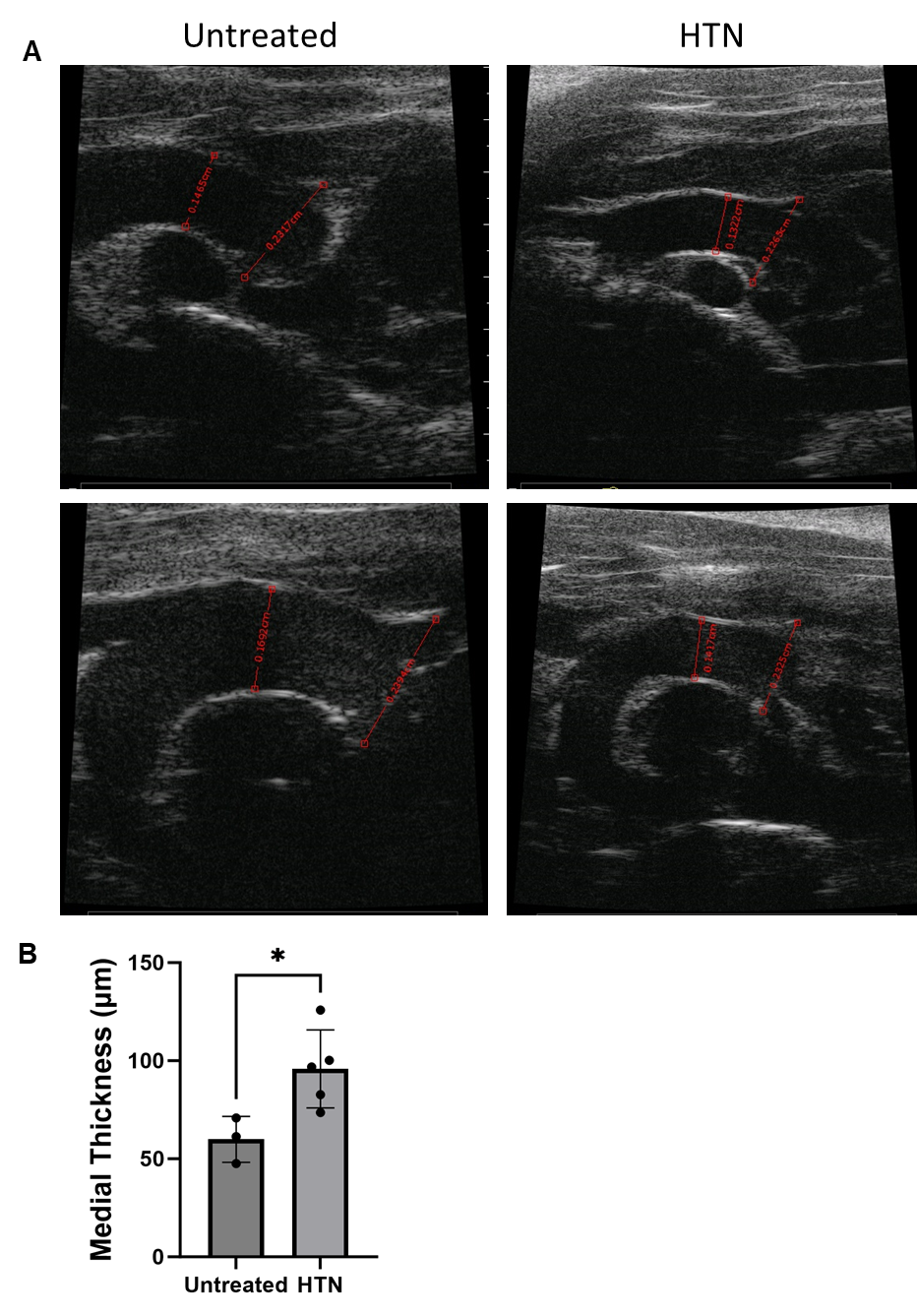
**

**Supplemental Figure 2** A) Representative echo images showing no dilation of the aorta in mutant mice at 6 months of age (after 4 months of HTN). Note: zoom is not standardized for all images. These images are examples matching the data in Figure 1D. B) Quantification of HE stained sections shows a small but significant increase in the thickness of the medial layer.


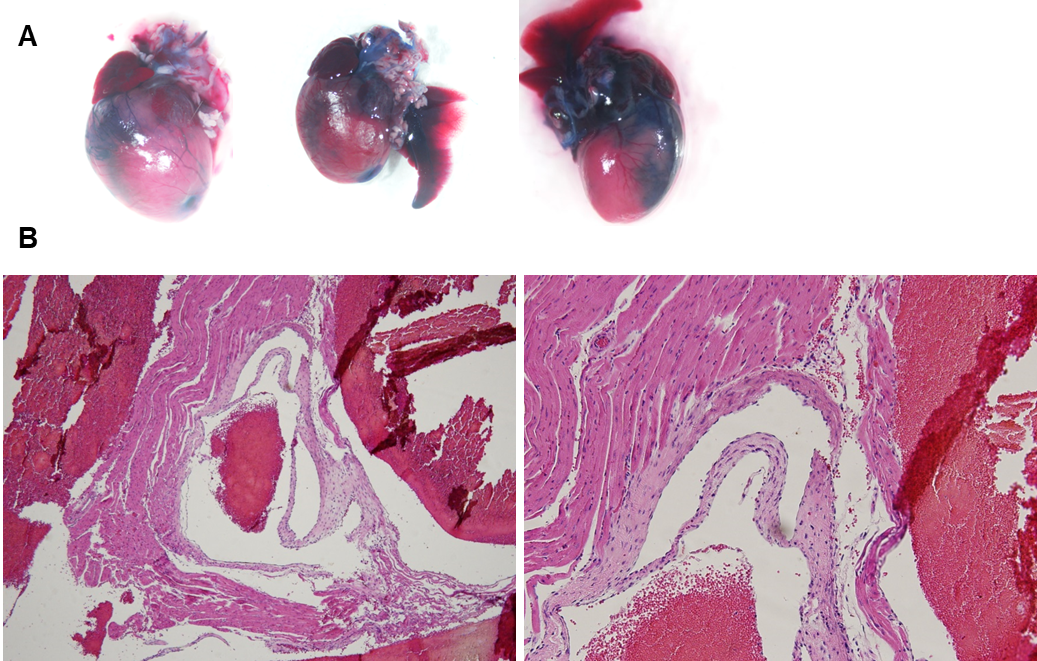


**Supplemental Figure 3** A) Gross images showing lack of aortic enlargement and pericardial tamponade in *Myh11*^R247C/R247C^ mice that died after NE injection. B) Histology sections from one *Myh11*^R247C/R247C^ mouse that died after NE injection shows a tear in the aortic root (zoomed in image on the right).

**
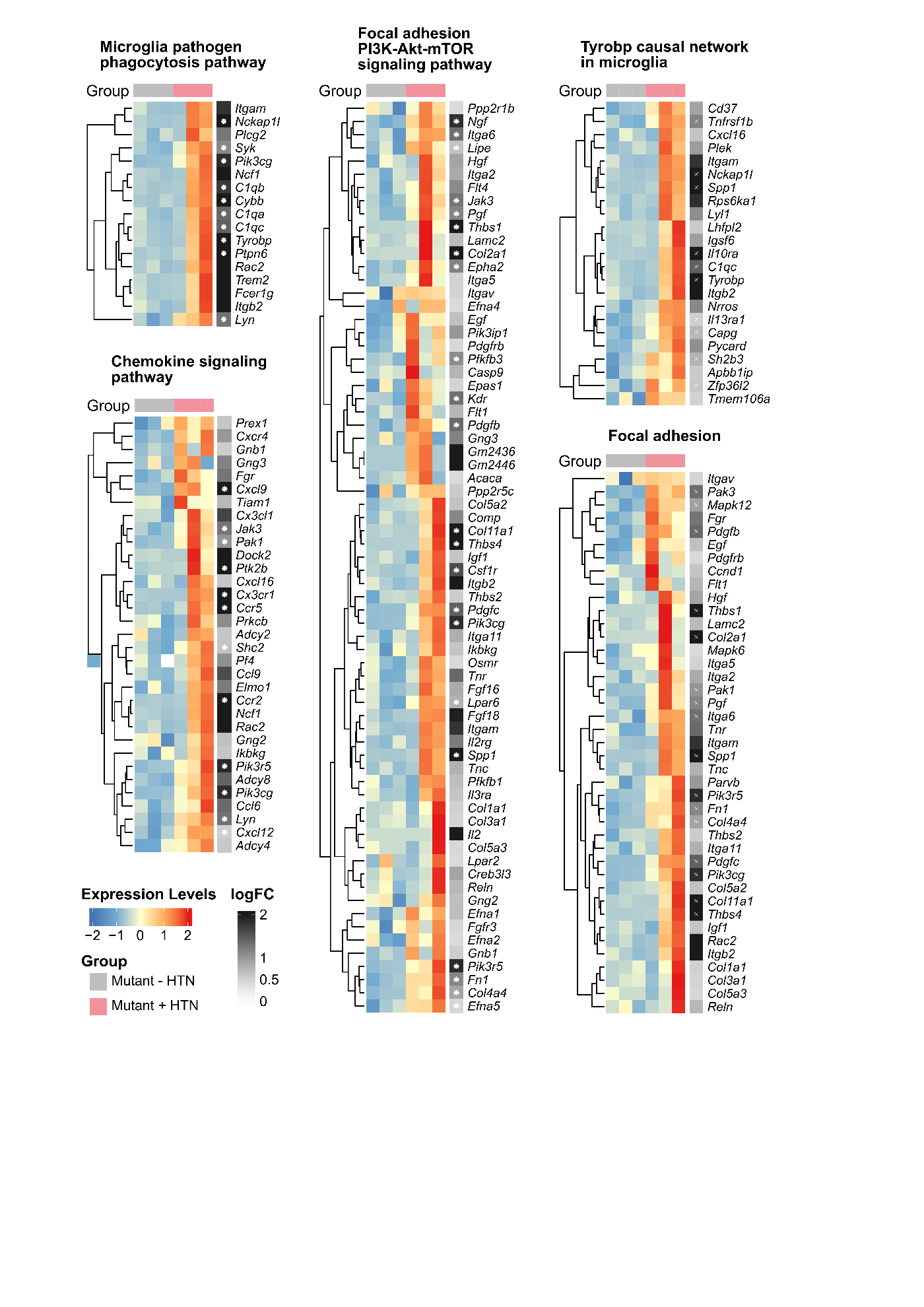
**

**Supplemental Figure 4.** Heatmap showing genes in each of the upregulated pathways shown in Figure 2B. These heatmaps represent the leading-edge subset of the enrichment analysis, and the greyscale representation on the right uses stars to label significant genes with FDR<0.05.
